# Transcriptome-wide analysis of the *Trypanosoma cruzi* proliferative cycle identifies the periodically expressed mRNAs and their multiple levels of control

**DOI:** 10.1371/journal.pone.0188441

**Published:** 2017-11-28

**Authors:** Santiago Chávez, Guillermo Eastman, Pablo Smircich, Lorena Lourdes Becco, Carolina Oliveira-Rizzo, Rafael Fort, Mariana Potenza, Beatriz Garat, José Roberto Sotelo-Silveira, María Ana Duhagon

**Affiliations:** 1 Laboratory of Molecular Interactions, School of Sciences, Universidad de la República, Montevideo, Uruguay; 2 Department of Genetics, School of Medicine, Universidad de la República, Montevideo, Uruguay; 3 Department of Genomics, Instituto de Investigaciones Biológicas Clemente Estable, Montevideo, Uruguay; 4 Institute for Research in Genetic Engineering and Molecular Biology 'Dr. N.H. Torres', Buenos Aires, Argentina; 5 Department of Cell and Molecular Biology, School of Sciences, Universidad de la República, Montevideo, Uruguay; Wageningen UR Livestock Research, NETHERLANDS

## Abstract

*Trypanosoma cruzi* is the protozoan parasite causing American trypanosomiasis or Chagas disease, a neglected parasitosis with important human health impact in Latin America. The efficacy of current therapy is limited, and its toxicity is high. Since parasite proliferation is a fundamental target for rational drug design, we sought to progress into its understanding by applying a genome-wide approach. Treating a TcI linage strain with hydroxyurea, we isolated epimastigotes in late G1, S and G2/M cell cycle stages at 70% purity. The sequencing of each phase identified 305 stage-specific transcripts (1.5-fold change, p≤0.01), coding for conserved cell cycle regulated proteins and numerous proteins whose cell cycle dependence has not been recognized before. Comparisons with the parasite *T*. *brucei* and the human host reveal important differences. The meta-analysis of *T*. *cruzi* transcriptomic and ribonomic data indicates that cell cycle regulated mRNAs are subject to sub-cellular compartmentalization. Compositional and structural biases of these genes- including CAI, GC content, UTR length, and polycistron position- may contribute to their regulation. To discover nucleotide motifs responsible for the co-regulation of cell cycle regulated genes, we looked for overrepresented motifs at their UTRs and found a variant of the cell cycle sequence motif at the 3' UTR of most of the S and G2 stage genes. We additionally identified hairpin structures at the 5' UTRs of a high proportion of the transcripts, suggesting that periodic gene expression might also rely on translation initiation in *T*. *cruzi*. In summary, we report a comprehensive list of *T*. *cruzi* cell cycle regulated genes, including many previously unstudied proteins, we show evidence favoring a multi-step control of their expression, and we identify mRNA motifs that may mediate their regulation. Our results provide novel information of the *T*. *cruzi* proliferative proteins and the integrated levels of their gene expression control.

## Introduction

*T*. *cruzi* is the causative agent of Chagas disease, also known as American trypanosomiasis, a parasitosis that affects more than 8–10 million people in the endemic areas of 21 Latin American countries [1]. Current pharmacological treatment relies on benznidazole and nifurtimox, drugs with low efficacy and high toxicity, which leads to the need for the development of improved compounds against Chagas disease [2]. In the post-genomic era, both genomic and transcriptomic information can be used to discover new pathogen specific drug-targetable proteins essential for the replication of the parasite [3].

The eukaryotic cell cycle is a coordinated sequence of stages consisting of periods of cell growth (G1-phase), DNA and organelle replication (S-phase), rapid cell growth and preparation for cell division (G2-phase), organelle segregation (M) and cell division (Cytokinesis). Accurate cell cycle progression is driven by a complex network of regulatory proteins that ensures the appropriate order of the phases and their proper initiation and completion. The cellular events taking place along the cell cycle are carried out by process-specific molecular machineries. Proliferation in trypanosomatids is expected to be particularly complex due to their highly polarized cell architecture and the presence of single copy organelles, including a large mitochondrion with a genome divided in multiple DNA circles and a flagellum, both connected through cytoskeletal filaments [4, 5]. Additional complexity to the control of replication is given by the existence of one proliferative stage at the insect vector (epimastigote), an intracellular proliferative stage in the human host (amastigote), and a non-dividing and infective stage (trypomastigote) [6]. Further differences in the canonical cell division, including a closed mitosis, the absence of centrioles, and the existence of the kinetoplast, account for substantial divergence in the mechanisms of proliferation of trypanosomatids in comparison to their mammalian hosts. This has led to the proposal of the protozoan parasite cell cycle as a relevant target for drug development against the diseases [7–10]. Thus, the discovery of the proteins that are responsible for the control and progression of the replicative cycle in *T*. *cruzi*, as well as those necessary for the acquisition of infectivity, has been foreseen as an essential step for the development of rational drug design [11, 12].

Most of the knowledge of the trypanosomatids cell cycle has been gained from *T*. *brucei*, the causative agent of the African trypanosomiasis (reviewed by Zhou *et al*.) [13]. Although *T*. *brucei* and *T*. *cruzi* share many characteristics, they are separated by a long evolutionary distance, which accounts for their striking differences in biology and pathology. The major events of the *T*. *cruzi* cell cycle (including morphological changes, organelle dynamics, structural reorganization of these processes) have been described mostly for the non-infective epimastigote stage of *T*. *cruzi* [5]. In addition, several studies on individual proteins regulated during the cell cycle have been reported. In particular, the DNA replication machinery has been partially characterized, and was shown to be different from those in higher eukaryotes in terms of both components and regulation [14, 15]. Histone mRNA and protein variations along the proliferative and developmental cycle, as well as several histone modifications, have been also reported [16–18]. Additionally, of the 10 cyclins annotated in the *T*. *cruzi* genome, only TcCYC2 and TcCYC6 have been studied and proven to be involved in cell cycle control [19, 20]. The same group has investigated the role of cyclin dependent kinases CRK1, and CRK3 and the regulatory subunit Tcp12^CKS1^, reporting that the first two might be bona fide cdk homologues with roles in cell cycle progression [21–23]. Another focus of intense study is the mechanism of replication of the unique trypanosome mitochondrion, the kinetoplast, as well as the proteins involved [24].

Regulation of gene expression throughout the cell cycle has been addressed in several organisms both at the level of individual genes as well as in a genome-wide manner (bacteria, budding yeast, mouse, and human cells, for a review see reference [25]). In higher eukaryotes, periodical gene transcription is achieved via sequential expression of two transcriptional programs driven by the well-characterized transcription factors E2F1 at G1/S-phase and the FOXM1 at mitosis in mammals, and orthologous genes in distant eukaryotes [26, 27]. However, the absence of transcription initiation control that has been largely demonstrated in trypanosomatids [28] suggests that unique posttranscriptional mechanism(s) may substitute for the transcriptional control that is prevalent in other eukaryotes; a fact that may indeed point to a divergent regulatory strategy in the parasite compared to the human host. Although the mRNA level of cell cycle regulated transcripts has been shown to be affected by their rate of degradation in yeast, there are very scarce studies at this level [29]. Despite the clinical relevance of the parasite cell cycle, only one cell cycle transcriptome has been published in trypanosomatids so far: the transcriptome of the procyclic form of *T*. *brucei* [30]. In *T*. *cruzi*, there are few reports of cell cycle regulated genes and we still lack a global view of the transcriptome remodeling throughout the cell cycle.

Here we present the cell cycle transcriptome of *T*. *cruzi* epimastigotes obtained by deep sequencing of poly-adenylated RNAs. We define the differentially expressed genes, we characterize their biological properties, and we identify possible regulatory mechanisms that might be used to regulate groups of RNA transcripts along the cell cycle of the parasite.

## Materials and methods

### Parasite cultures, hydrouxyurea-induced synchronization and flow cytometry analysis

A *Trypanosoma cruzi* unnamed strain belonging to the TcI linage (DTU), as typified by multiplex Real-Time PCR assay [31], was selected for this study based on its ability to synchronize using the available hydroxyurea (HU) protocols, as previously shown by Potenza *et al*. [19]. Epimastigotes were grown at 28°C in liver infusion tryptose medium (LIT) supplemented with 10% heat-inactivated fetal bovine serum (FBS, from Capricorn Scientific GmbH). Parasites were synchronized with HU as previously described [32]. Briefly, early exponential-phase cultures (4 x 10^6^ parasites/mL) were transferred to fresh LIT medium containing 20 mM HU for 24 h. HU was then removed from the medium by centrifugation (1200g 5 min), the cells were washed twice in cold phosphate-buffered saline (PBS) and resuspended in fresh LIT medium. To obtain G1, S and G2/M enriched parasite populations, samples were collected at 0, 6 and 13 h post-HU release respectively. A sample of parasites harvested before the incubation with HU was used as a control of asynchronous growth. For each time-point studied, an aliquot of 2 x 10^6^ parasites/mL was washed twice in cold-PBS and fixed overnight at 4°C in 500 μL 70% Ethanol-PBS. Propidium iodide (PI) staining was carried out by incubation of the fixed parasites for 30 min at 37°C in PBS containing 20 μg/mL PI and 200 μg/mL RNAse A for DNA-specific staining. Three technical replicates per biological sample were analyzed for DNA-content in a Flow cytometer (FACSCalibur, BD Biosciences) and the proportion of G1, S and G2/M cells in the samples was determined as previously described [33].

### RNA isolation and RT-qPCR analysis

Total RNA was obtained using TRIzol® Reagent (Invitrogen™), according to manufacturer's instructions. The obtained RNA was treated with DNAse, according to manufacturer’s protocol (DNA-free, Ambion) and quantified in a Nanodrop spectrometer (Thermo Scientific, USA). cDNA was synthetized from 1 μg of total RNA using Superscript™ III first strand synthesis kit (Invitrogen™) and random primers. The mRNA levels were determined by amplification of the cDNA in a StepOne® (Applied Biosystem™) real-time PCR analyzer using CDS-specific primers. The oligonucleotides were designed to match the sequence of the strain used in this study, which was obtained by RNA-seq. Relative amounts of the target genes were normalized to the TcTub (beta-tubulin) gene, and changes in gene expression were determined by the 2^–ΔΔCt^ method [34].

### Library preparation and RNA-sequencing

Total RNA of synchronized epimastigote cultures was extracted with Trizol® Reagent (Invitrogen™) and treated with DNAse as explained above. Equimolar amounts of RNA derived from the three independent synchronizations (biological replicates) were pooled. Poly-A+ mRNAs were isolated by oligo-dT selection and single-end 50 bp sequencing libraries for RNA-seq analysis were built on standard Illumina RNA-seq protocols. Sequencing experiments were performed on an Illumina® HiSeq™ 2000 platform (service provided by BGI Americas). Raw reads obtained were subjected to a data filtering method by standard protocols performed by the sequencing service provider, which consisted in the removal of adapter sequences, contamination and low-quality reads from the dataset. The raw sequence data produced in this study was deposited at the NCBI-SRA [BioProject: PRJNA310212].

### Mapping and gene quantification analysis

High quality reads were mapped to the Esmeraldo haplotype of the CL-Brenner genome (version 4.2, downloaded from tritrypdb.org) [35] using bowtie2 [36] with default ‘—very-sensitive-local’ parameters. Using the gene annotation file (gff file, downloaded from tritrypdb.org), reads mapping to RNA features were counted with HTseq version 0.6.0 [37] with the default “union” mode. Only reads with a mapping quality better than 10 were considered for gene expression analyses. Reads mapping to non-coding RNA features were set apart from the dataset. Gene expression data was derived from the gene counts normalized for sequencing depth using the DESeq package (version 1.18.0, implemented in R statistical environment) [38] yielding “normalized gene counts” (nCounts), expressed as reads per kilobase (RPK).

### Differential gene expression and gene ontology (GO) analysis

To assess differential gene expression (DEG), the normalized gene counts were used as an input in the negative binomial distribution test of the DESeq package [38]. Since the three replicates used in this study were pooled and sequenced together, the method “blind” and the sharingMode “fit-only” using the three cell cycle phases sequenced were applied to estimate dispersions. From these calculations, cell-cycle regulated genes were defined as genes with a fold change greater than 1.5, supported by a p-value lower than 0.01. The lists of differentially expressed genes were analyzed for enrichment of GO terms using the online analysis tool available at *tritrypdb*.*com*, a feature that implements a Fisher’s exact test. A p-value lower than 0.01 was used as a threshold for significant term overrepresentation. Principal Component Analysis (PCA) was performed with *ClustVis* web tool [39], using RT-qPCR and RNA-Seq data for 21 DEGs, normalized by Tubulin gene expression (S2 Table). To compare the two types of data (Cts and reads), phase specific gene expression was transformed into a proportion of the total additive expression of the three stages (*i*.*e*. each gene is represented by three fractions, one per stage, which together add to 1).

### Comparisons of *T*. *cruzi* cell cycle regulated genes in other eukaryotes

The non-redundant *T*. *cruzi* orthologue genes present in *T*. *brucei* TREU 927 were obtained from *tritrypdb*.*org* using the "Transform by Orthology" online tool. For the identified orthologues, we compared their peaking times in the *T*. *brucei* cell cycle transcriptome [30] to those identified in the current study. In order to draw a comparison with the human and yeast cell-cycle studies, the putative orthologue genes were determined by one-way blast (tBLASTx), filtering the results with cutoffs of 1E-4 E-value and 50 bit-Score. *Cyclebase* was taken as the reference database for cell-cycle gene expression, then the lists of periodically regulated genes were retrieved from the website *cyclebase*.*org* [25]. *T*. *cruzi* orthologues for human and the budding yeast genes were identified and coincidences were counted. The proportion of orthologues was assessed for all expressed genes (7860 genes dataset) and for the cell cycle regulated genes (305 genes dataset).

### Determination of 5’ and 3’ untranslated regions (UTRs)

For the determination of the UTRs, reads that contained the mini-exon sequence 5’- AACGCTATTATTGATACAGTTTCTGTACTATATTG-3’ (ME), or the poly-A sequence 3’-AAAAAAAAAAAAAAAAAAAA-3’ (PA) were extracted from the dataset and the ME or PA sequence was trimmed using *cutadapt* (-g -m 20 -O 8 parameters were set). The remaining reads were mapped to the reference Esmeraldo-like CL-Brenner genome using Bowtie2 default ‘—very-sensitive-local’ parameters. Only uniquely mapping reads with a mapping quality greater than 0 were considered. In order to avoid false trans-splicing calls, the genomic region upstream of the mapping position was evaluated for homology with the ME sequence using an in-house python script. Finally, non-identical reads, whose mapping corresponded to the same ME trans-splicing or polyadenylation site, were collapsed and counted. Only UTRs with a minimum of 2 non-identical reads were considered. Extremely large UTRs that contained stretches of at least 100 Ns in the genomic sequence were removed from the dataset. Finally, for each gene, the UTR represented by the most reads was selected and used to compile a database of empirically determined *T*. *cruzi* epimastigote UTRs.

### Definition of putative transcription start sites and gene positioning analysis

Genomic position and strand information were obtained for each gene from the annotation file (gff file) downloaded from *tritrypdb*.*org*. Putative polycistronic units were defined as a continuous group of at least two mRNA coding genes encoded on the same strand, as previously done [40]. Single non-coding RNA genes were considered as independent transcribed units, since polycistronic transcription is interrupted by these genes [41]. The transcription start sites (TSSs) were defined as the midpoint of the strand switch regions; for the telomeric polycistron, the TSSs were defined as the midpoint between the start of the polycistronic unit and the next chromosome end. Finally, the distance between each gene and the nearest equally-oriented TSS was calculated. Python scripts were designed to perform the analyses described above.

### Determination of codon adaptation index

The relative codon usage was estimated from the frequency of a given codon within the synonym codon group, using the software packages GCUA (General Codon Usage Analysis)[42] and INCA (INteractive Codon usage Analysis) [43]. The codon adaptation index (abbreviated as CAI) of a particular gene is a measurement of the deviation in the codon usage from the codon usage of a reference group [44]. Ribosomal proteins were used as the reference group, thus CAI varies from 0 to 1, where 1 is the optimal adaptation equivalent to that of the ribosomal proteins. The GC-content of a sequence (G+C), defined as the percentage of GC bases, and the GC-content in the third codon position (GC3) of the coding region were calculated for every gene using in-house python scripts.

### Clustering and motif identification

The gene expression values for the cell cycle regulated genes were clustered by one-minus Pearson correlation using the GENE-E software (available at www.broadinstitute.org/cancer/software/GENE-E/) to obtain groups of putative co-regulated genes. For these groups, the UTR sequences were obtained from *tritrypdb*.*org* by the sequence retrieval tool as the 300 bp downstream of the STOP codon and 100 bp upstream of the AUG codon for the 3’ UTR and 5’ UTR respectively. The DREME tool [45] from the MEME suite [46] was used to discover short ungapped motifs that are relatively enriched in the UTRs sequences of the regulated genes compared with control UTR sequences from the non-regulated genes. Only motifs on the transcribed strand with E-values greater than 0.05 were considered. The structural motifs were identified using the *Infernal* package [47] on sequences retrieved from *tritrypdb*.*org*, defined as the 450 bp downstream of the STOP codon for the 3’-UTRs, and the 150 bp upstream and 50 bp downstream of the AUG codon for the 5’-UTRs. For the discovery of the motifs, the algorithm *cmfinder* [48] was used with the following parameters: “−s1 −f 0.4−c10”. A further assessment of the quality of the identified motif was sought by calculating a sensitivity and specificity value. Sensitivity was assessed using the algorithm *cmsearch*, and is defined as the percentage of sequences found when using the consensus motif as a query in the total of sequences that supported the motif at the discovery stage. Specificity was calculated through *cmsearch* by using the motif to query a series of random groups with the same number of genes as the discovery group, where every hit was considered a false positive; specificity was calculated as 1 minus the frequency of total false positive hits. The *RNAfold* algorithm of the *ViennaRNA* package 2.0 [49] was used to calculate the free energy of the consensus motif.

## Results

### Isolation of cell cycle stages using HU synchronization and RNA preparation

**Fig 1:**
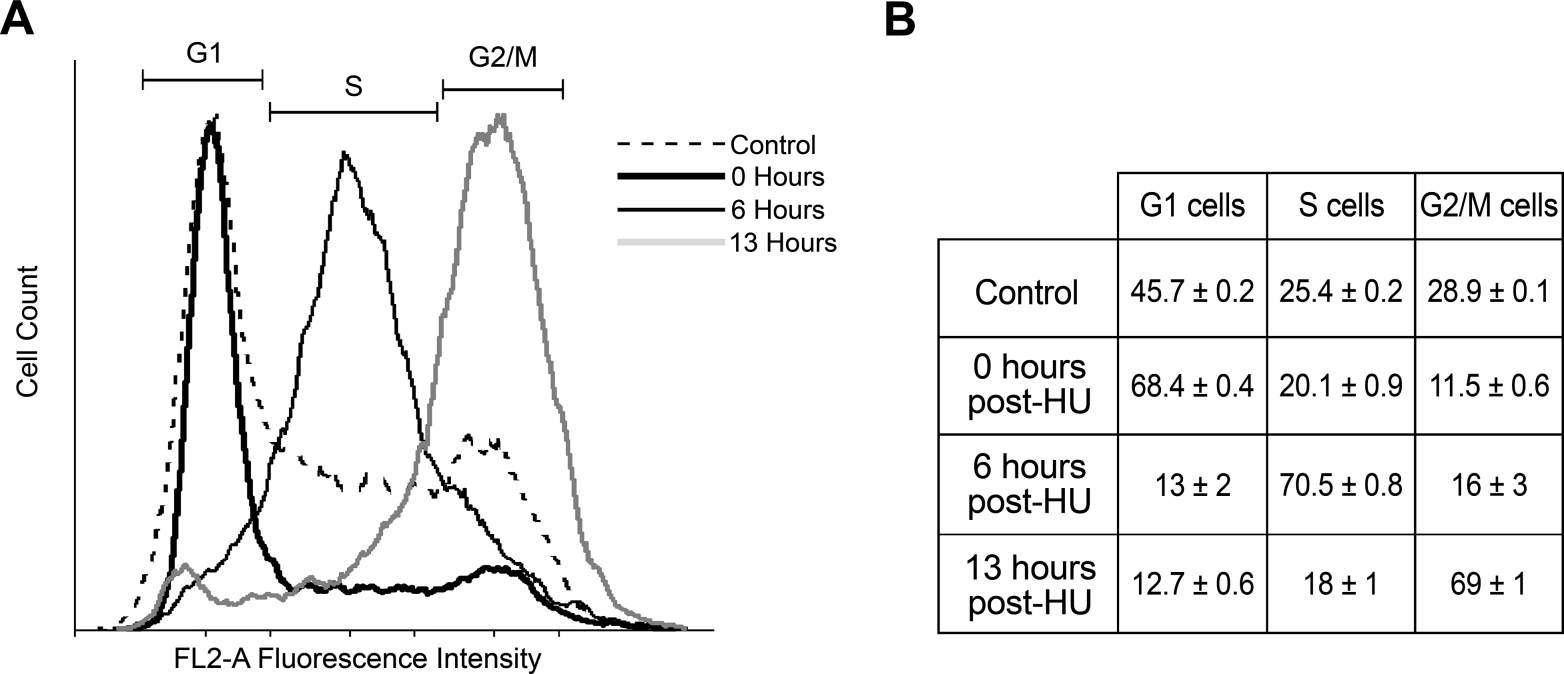
Cell cycle phase distribution of synchronized *T*. *cruzi* epimastigotes. DNA content analyses using propidium iodide staining were performed for parasites populations at 0 h (bold line), 6 h (simple line), and 13 h (grey line) post-HU release, which correspond to G1, S, and G2 peaking times respectively. Representative experiments of each phase are presented (solid lines), and an asynchronous culture harvested prior to HU treatment is shown as a control (dotted line). The inset table shows the percentages of cells gated in G1, S or G2/M-phase for the three replicates, expressed as mean ± SEM.

In order to obtain parasite cultures synchronized at different cell cycle stages, we carried out a HU exposure protocol described previously [32], using *T*. *cruzi* epimastigote cultures of an unnamed TcI lineage strain. Due to the variability in the HU response among the different strains [33], the choice of strain for our study was based on cell synchronization efficiency [19]. DNA-content was analyzed by flow cytometry to assess the efficiency of the synchronization protocol. We determined that 0, 6 and 13 hours post HU-washout were the optimal collection times to obtain G1, S and G2/M populations respectively, which allowed for a maximum cell cycle phase enrichment of approximately 70% (Fig 1). The small standard error of the cell cycle phase enrichment observed among the synchronization replicates (between 0.4–1%) supports the reproducibility of the method.

### Deep sequencing of the RNA purified from epimastigotes synchronized at different cell cycle stages

In view of the high reproducibility of the cell synchronization protocol, total RNA of three independent synchronization experiments (replicates) were pooled in equimolar concentrations for the generation of single RNA samples representative of each individual cell cycle stage (G1, S G2/M). Given their advantage for sequencing cost reduction, pools of sample replicates have been used in RNA-seq studies, and their performance has been extensively discussed [50–52]. One of the main caveats of the pooling approach is a potential decrease in detection power, which can be partially compensated by increasing the sequencing depth [53]. Therefore, we designed a sequencing protocol intended to obtain very deep transcriptome data. In addition, we applied DESeq to our study, since in the absence of independent replicates DESeq has been shown to produce the most conservative determination of DEGs in comparison to other methods [54]. Following this approach, we prepared one library for each RNA pool of triplicate samples for each cell cycle stage (G1, S and G2-M) and sequenced them using standard Illumina poly-A+ RNA-seq protocols. Over 44 million high-quality 50 bp single-end reads per library were produced (S1 Table), of which more than 32 million reads per sample were mapped to the reference Esmeraldo-like CL-Brenner genome, representing an alignment proportion of 72%. Due to the highly repetitive nature of the *T*. *cruzi* genome [11], only uniquely mapping reads were used for gene expression analysis; these comprise over 8 million reads mapping to annotated transcripts, which embodies 27–28% of the total mapped reads.

Then, the expression value for each gene was calculated using DESeq as the relative number of reads mapping to the transcript in the total reads, and expressed as normalized read counts (nCounts). A list of DEGs was established for the three cell cycle transitions isolated (G1 to S, S to G2/M, and G2/M to G1) by comparing the nCounts obtained in different phases. In order to select those transcripts whose quantification was independent of the sequencing depth, the amplitude of expression was calculated as in Archer *et al*. [30] and plotted against the sequencing depth (S1 Fig). We found that transcripts with more than 100 nCounts have small and constant amplitudes in the three different libraries, thus we set this value as the minimum requirement for gene inclusion in global transcriptome analysis. That resulted in a dataset of 7860 genes, representing 76% of the protein coding genes annotated in the genome of *T*. *cruzi* Esmeraldo haplotype of the CL-Brenner genome. This sequencing depth is very high for a cross-strain mapping in *T*. *cruzi* (Tcl vs. CL Brenner Esmeraldo-like), as is seen by the comparison with a recent high resolution transcriptome that achieved 56% mapping in epimastigotes using a *T*. *cruzi* Y strain [55].

**Fig 2:**
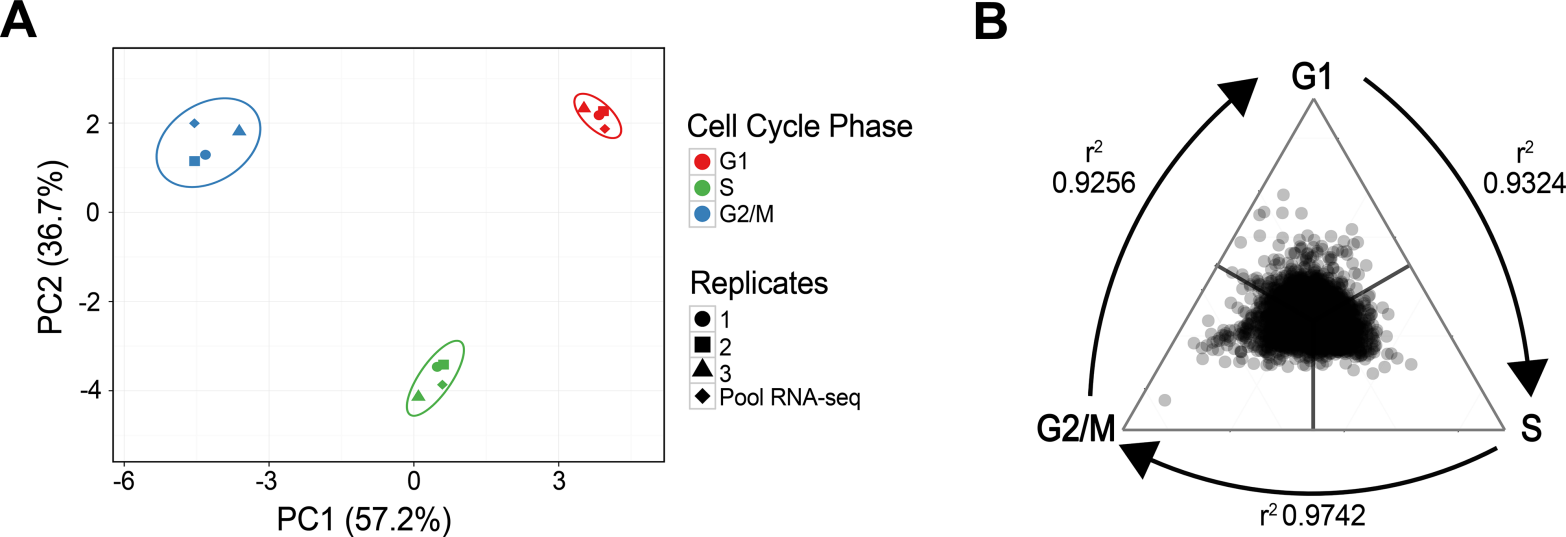
Comparative analysis of the samples and the resulting RNA-sequencing datasets. A. Principal Component Analysis of the individual replicates (ΔΔCt values relative to tubulin, obtained by RT-qPCR) and the pooled samples used for sequencing (nCounts normalized by tubulin, obtained by RNA-Seq), based on the quantification of 21 selected differentially expressed genes (see S2 Table). B. Phase specific gene expression values compared in a ternary plot. Gene expression was denoted as the gene expression value in each cell cycle stage relative to the total additive expression of the three stages. Thus, each gene is represented by three fractions, one per stage, which together adds 1. The values were plotted in R using the ggtern library. Pearson correlation coefficients (r^2^) were calculated for every set of 2 cell cycle phases and are presented next to the corresponding connecting arrow.

Due to the use of pooled RNAs, we first carried out an assessment of putative outlier samples among the replicates. For that purpose, we performed RT-qPCR of individual replicates for 21 genes that showed differential expression during the cell cycle (S2 Table). The relative gene expression data obtained was analyzed in a principal component analysis (PCA) together with the same data extracted from the transcriptome of the pools (Fig 2A). The two principal components obtained by PCA, which explain 93,9% of the variation, clearly segregate together the three cell cycle phases, independently of the method (RNA-seq vs. qPCR) and sample (individual replicates vs. pools) used. In addition, no replicate shows a conspicuous deviation from the clusters. As a complementary approach, we compared every sample using Pearson correlation coefficients and found out that the similarity among the samples is attributable to the specific cell cycle phase (S3 Table). Altogether, both PCA and correlation analysis exclude the presence of outlier samples in this dataset.

In order to assess the extent of the global changes in mRNA abundance along the cell cycle, we calculated the correlation of the expression of the 7860 genes between the three phase specific datasets (Fig 2B). We observed r^2^ Pearson correlation coefficients above 0.92 for the three transitions studied, which indicates that the transcriptome is predominantly stable during these cell cycle transitions. Nevertheless, distinctive degrees of regulation were detected; for instance, the higher correlation observed between the S and G2/M profiles in comparison with any of these two phases relative to the G1-phase indicates that G1 gene expression is the most divergent of the three.

### Analysis of the epimastigote cell cycle regulated genes

We then focused on the study of the subset of genes differentially expressed along the epimastigote cell cycle. For that purpose, we obtained a list of differentially expressed genes for the three cell cycle transitions isolated. Using an amplitude greater than 1.5 as a threshold, we found that 7185 genes (91%) have stable mRNA levels in all three phases. Among the remaining 675 genes (9%), 305 genes reached a *p*-value lower than 0.01 as calculated by DESeq (see S4 Table for the complete list of genes). The latter genes were therefore defined as the cell cycle regulated genes in this study, and are also referred as periodically expressed genes. The 305 cell cycle regulated transcripts display a wide range of abundance, showing an average of 1227 nCounts/phase, corresponding to a median of 3702 total gene reads.

**Fig 3:**
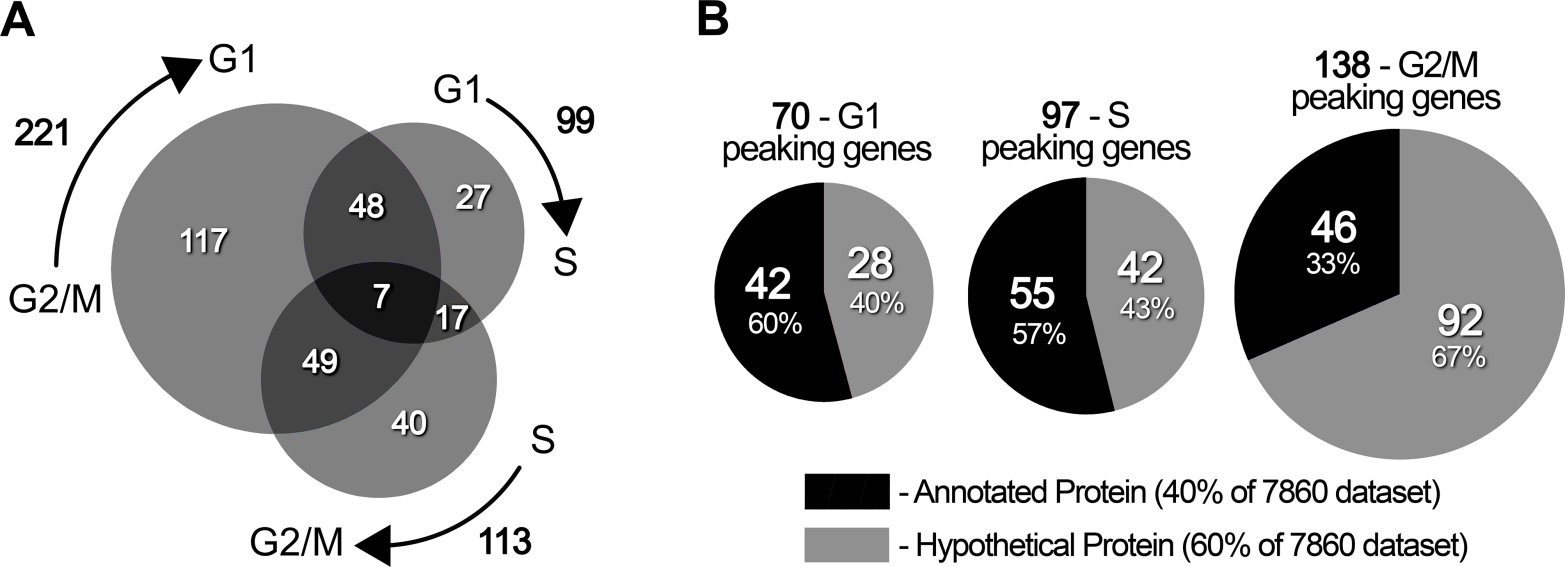
Differential gene expression throughout the *T*. *cruzi* epimastigote cell cycle. A. Transcript modulation along the three phase transitions studied. The three circles of the Venn diagram represent the genes significantly modulated in each of the three phase transitions: G1-S, S-G2/M and G2/M-G1 (amplitude values higher than 1.5 and p<0.01). The total number of genes modulated are indicated outside the arrows, whereas the numbers inside the circles indicate the amount of common as well as transition specifically modulated genes. The plot was generated using the on-line application *BioVenn* [56]. B. Proportion of hypothetical and annotated genes in each phase. The peaking genes were counted for each of the cell cycle phases and the number of annotated versus hypothetical proteins was plotted for each group of genes. *TritrypDB* gene names were consulted to address the hypothetical annotation of the proteins.

In terms of gene number, we found that the most regulated cell cycle transition was G2/M to G1, as represented by the largest circle in the Venn diagram of Fig 3A; it accounts for a total of 221 modulated genes. The largest variation in gene number seen in this transition goes in agreement with its lowest r^2^ Pearson correlation among the gene sets (Fig 2B). For the S to G2/M and the G1 to S transition we detected 113 and 99 differentially expressed genes respectively. Among these, 184 genes were regulated only in one transition, while 121 were implicated in two transitions and only 7 genes were found to be regulated in the three of them (Fig 3A).

In view of the important proportion of genes that vary their abundance in more than one cell cycle phase transition, we decided to describe differential gene expression in an alternative way; we categorized the genes based on the phase in which they show the highest expression values, thus we later refer to them as phase specific “peaking” genes. Following this approach, we identified 70, 97 and 138 genes that peaked at G1, S and G2/M-phases respectively (Fig 3B). When we looked at the proportion of hypothetical proteins within each phase, we noticed an enrichment of annotated genes at both G1 and S (60 and 57% annotated proteins respectively) in comparison with G2/M (43% annotated proteins). Since the control group of 7860 expressed genes had 40% annotated proteins, G1 and S proteins are probably more similar in sequence to characterized eukaryotic proteins than the average *T*. *cruzi* proteins. In order to validate the RNA-Seq approach, we used RT-qPCR as an independent quantification method. We selected 21 DEGs, expressed at high, low, and intermediate levels, as determined by the RNA-Seq. The results show a very small deviation among the three replicates for every individual gene and a high correlation among all the changes measured by qPCR and RNA-Seq (S2 Fig).

To explore the functions of the identified cell-cycle regulated genes, the GO terms of the peaking genes in each of the cell cycle stages were studied (see Table 1 and S5 Table for the complete list of terms). For the 70 genes that peaked in the G1-phase, cellular functions related to the metabolism of carbohydrates and energy production were overrepresented. In addition, three putative chaperons (hsp70 and DNAJ like), four peptidases, one kinesin, one tyrosine ligase, and one ubiquitin carrier protein peaked at G1. The list also comprises a cyclin-2, a CDC2 kinase and a Cyclin A/CDK2 associated like-proteins. Finally, six factors related to DNA modification (topoisomerase, polymerase I, exonuclease, nucleoplasmin, kDNA associated protein), three involved in nucleotide metabolism, and an RNA helicase are also present.

**Table 1:** GO Enrichment Analysis for the cell cycle phase-specific peaking genes.

Phase	GO group	GO Term	Gene count	Fold enrichment	Odds ratio	P-value
**G1**	MF	carbohydrate kinase activity	3	34.3	36.5	5.7E-03
**G1**	BP	carbohydrate metabolic process	7	6.5	7.5	6.7E-03
**G1**	CC	glycosome	4	9.4	10.1	8.8E-03
**G1**	BP	hexose metabolic process	5	9.9	11.0	1.1E-02
**G1**	BP	monosaccharide metabolic process	5	9.5	10.5	1.4E-02
**G1**	CC	peroxisome	4	8.1	8.8	1.4E-02
**G1**	CC	microbody	4	8.1	8.8	1.4E-02
**G1**	MF	galactokinase activity	2	91.4	95.3	2.1E-02
**G1**	BP	cellular carbohydrate metabolic process	4	12.6	13.7	2.3E-02
**G1**	BP	generation of precursor metabolites and energy	5	8.3	9.2	2.5E-02
**S**	BP	DNA conformation change	7	13.6	15.2	1.8E-04
**S**	BP	DNA metabolic process	12	5.4	6.5	2.8E-04
**S**	BP	DNA packaging	6	14.4	15.8	7.9E-04
**S**	CC	kinetoplast	4	28.8	30.7	8.4E-04
**S**	BP	DNA replication	7	9.7	10.8	1.4E-03
**S**	MF	DNA binding	11	4.1	4.8	2.5E-03
**S**	BP	nitrogen compound metabolic process	23	2.3	3.1	7.4E-03
**S**	CC	mitochondrion	13	3.0	3.6	9.4E-03
**S**	BP	nucleobase-containing compound metabolic process	22	2.3	3.0	1.1E-02
**S**	CC	intracellular membrane-bounded organelle	23	2.0	2.6	1.3E-02
**G2/M**	MF	motor activity	10	8.0	9.5	1.8E-05
**G2/M**	MF	microtubule motor activity	9	9.0	10.4	3.1E-05
**G2/M**	BP	microtubule-based movement	9	8.8	10.3	3.8E-05
**G2/M**	BP	microtubule-based process	10	7.2	8.5	5.2E-05
**G2/M**	BP	cellular component movement	9	7.2	8.4	1.7E-04
**G2/M**	MF	ATP binding	21	2.7	3.6	4.2E-04
**G2/M**	MF	adenyl ribonucleotide binding	21	2.7	3.6	4.3E-04
**G2/M**	MF	adenyl nucleotide binding	21	2.7	3.6	4.4E-04
**G2/M**	CC	cytoskeleton	9	5.1	5.8	1.7E-03
**G2/M**	CC	microtubule associated complex	7	7.0	7.8	1.8E-03

MF, Molecular function; BP, Biological process; CC, cellular component

A quite distinctive GO list is found for the 97 genes that peaked at S-phase, which, as expected, is enriched in proteins involved in DNA and chromatin replication and nucleotide metabolism. mRNAs of enzymes that participate in nuclear and mitochondrial DNA synthesis, corresponding to the proliferative cell nuclear antigen (PCNA), polymerase theta, mitochondrial DNA polymerase beta and DNA primase, DNA ligases and DNA topoisomerase IA, were upregulated. In addition, transcripts for proteins that organize chromatin, like histone H2A, H2A variant, H4 and high mobility group TcHMGB, increase their levels at this stage. Interestingly, the most upregulated transcript found in the 305-gene set is the kinetoplast associated protein KAP3, whose orthologue in *T*. *brucei* is a well characterized cell cycle dependent gene overexpressed during S phase [57]. Indeed, two other putative kinetoplast associated proteins are also upregulated. At this phase, an increase of three snRNA associated proteins (U6, U2, and Sm-F putative) is observed. We also found proteins with roles in translation, such as putative eukaryotic translation initiation factor 1A and a seryl-tRNA synthetase. A transcript for a cyclin regulatory subunit was also identified, as well as a wider spectrum of chaperons relative to G1-phase, composed of five types of chaperons (hsp85, hsp60, hsp70 and DNAJ like). Interestingly, two cyclophilins are shown to be upregulated at this point of the cell cycle. In addition, the preparation for the massive cytoskeleton reorganization that takes place at G2/M [58] may be supported by the upregulation of a putative cofilin/actin depolymerizing factor.

Finally, cellular functions related to the mitotic spindle formation and organelles organization were found overrepresented in the 138 genes that peaked in the G2/M-phase of the cell cycle. This is based on the overexpression of several mechanochemical proteins comprising 6 dynein, 6 kinesins and a probable myosin heavy chain. Besides, a *T*. *cruzi* orthologue of TbCPC2 chromosomal passenger gene is upregulated, together with transcripts encoding proteins involved in DNA conformation (helicase), repair (endonuclease, excision/repair) and packing proteins (histone and histone modifiers, chromosomal passenger), as well as kDNA binding proteins (UMSP and a putative kDNA associated proteins) and factors related to nucleotide metabolism. Two putative cyclins (CYC2-like and putative cyclin 6) and a cell division related protein kinase 2 transcript are also upregulated during this phase. The mRNA for the RNA binding protein PUF9 and for an RNA helicase increase their abundance in G2/M-phase. Among the protein modifying enzymes overrepresented in this phase there are six kinases, comprising one of the polo-like type and a putative dephospho-CoA kinase, two calpain cysteine peptidases, two phosphatases (tyrosine and Serine/threonine type) and a ubiquitin-conjugating enzyme.

### Comparison of the cell cycle regulated genes among species

The comparison of gene expression datasets is an invaluable tool to interpret and validate functional genomics data of a given biological condition. In order to apply this tool to the understanding of the *T*. *cruzi* cell cycle we performed a **metanalysis** of the 305-cell cycle regulated genes in other species. We firstly sought to analyze the similarities in the cell cycle regulated proteins between the two main trypanosomatid human parasites. Of the list of 305 regulated genes found in *T*. *cruzi*, we recognized 247 orthologues in the *T*. *brucei* genome, which implies that 58 *T*. *cruzi* cell cycle regulated genes (30%) diverge between the two pathogens (Table 2). In addition, we determined that *T*. *cruzi* periodically regulated genes have proportionally more orthologous genes in *T*. *brucei* genome than non-regulated genes (p-value 6.0 E-14, Fisher test). Then, we compared the *T*. *cruzi* cell cycle transcriptome obtained in this work with the one published in *T*. *brucei* [30]. In this study, the authors used elutriation to isolate procyclic parasites (the insect stage) in early G1, late G1, S and G2/M, and found a total of 546 cell cycle regulated genes. However, only 30% (75/247) of them have orthologous genes in the 305 *T*. *cruzi* cell cycle list, of which 39% (29/75) peaked in the exact cell cycle phase, whereas the other 61% peaked in the phase immediately earlier than the corresponding *T*. *cruzi* phase. The S phase presents the highest number of orthologues between the two organisms and the higher percentage of shared cell cycle genes.

**Table 2:** Comparison of *T*. *cruzi* and *T*. *brucei* cell cycle regulated genes.

	Cell-cycle regulated genes in *T*. *cruzi*		*T*. *cruzi* genes with *T*. *brucei* cell-cycle regulated orthologue		
PHASE	All	With *T*. *brucei* orthologues	All	Same-phase	Different-phase
G1	70	62	12 (19%)	12^a^	-
S	97	87	31(36%)	6	1 (Early G1) / 24 (Late G1)
G2/M	138	98	32 (33%)	11	5 (Late G1) / 16 (S)
All Reg	305	247	75 (30%)	29 (39%)	46 (61%)

^a^*T*. *brucei* G1 genes include the early and late G1 gene lists from Archer, *et al*.

Due to the therapeutic interest of the identification of parasite specific proliferative factors that are not present in the human host, we compared the *T*. *cruzi* periodically regulated transcripts across the two organisms. We first searched for genes homologous to the 305-cell cycle regulated transcripts on the human genome and we found 117 proteins (38% of periodical genes). Taking as reference the total number of homologous genes between the two species (3258 *T*. *cruzi* genes, i.e. 31% of total genes), we determined that *T*. *cruzi* periodically regulated genes have proportionally more homologous genes in the human genome than non-regulated genes (p-value 0.0102, Fisher test). This may indicate a slightly higher conservation of the proliferation related proteins between the two species, relative to other cell processes (1.36 odds ratio). We then compared the periodically expressed genes, using the already defined 600 cell cycle dependent human transcripts [59], available at the *Cyclebase* [25]. We found only 28 (24%) human homologues of the 305 *T*. *cruzi* gene set that are also periodically regulated at the level of transcript along the cell cycle.

### Assessment of the regulatory complexity of cell cycle genes by analysis of *T*. *cruzi* transcriptomes

**Nuclear-cytoplasmic** compartmentalization of RNAs is an active process that regulates genes expression through the control of the accessibility of the mRNAs to the protein synthesis machinery. We have recently addressed the sub-cellular distribution of RNAs in *T*. *cruzi* by sequencing the total, nuclear and cytosolic transcriptome of proliferative epimastigotes [60]. We found 444 and 738 transcripts more abundant in the cytoplasm and the nucleus respectively. Seeking to investigate if the 305-cell cycle regulated RNA transcripts are subject to compartmentalization, we determined how many are expected by chance in the nucleus or the cytosol and how many were identified in the dataset, and we then calculated the statistical enrichment observed (Table 3). The *T*. *cruzi* cell cycle dependent transcripts turned out to be enriched twofold in the cytosol of the epimastigotes (p-value 2.2E-03). This over-representation was found to be more significant for G1, which is the phase more abundant in an asynchronously dividing cell population. As anticipated from the previous observation, the cell cycle transcripts tend to be under-represented in the nucleus (0.9-fold enrichment (FE) and p-value (6.5E-01)).

**Table 3:** Analysis of cell cycle regulated genes in sub-cellular compartments.

	Up Cytoplasm		Up Nucleus	
Phase	O&(E)	FE (p-value)^a^	O&(E)	FE (p-value)^a^
G1	9 (3.1)	3.3 (2.9E-03)	3 (5.4)	0.6 (4.9E-01)
S	7 (4.3)	1.7 (2.0E-01)	3 (7.4)	0.4 (1.6E-01)
G2/M	9 (6.1)	1.6 (2.0E-01)	13 (10.5)	1.4 (3.1E-01)
All Reg	25 (13.3)	2.0 (2.2E-03)	19 (23.4)	0.9 (6.5E-01)

O&(E), observed and expected number of genes in each dataset; FE, Fold enrichment

^a^ Fold-enrichment and P-values obtained from Fisher contingency test.

As an additional strategy to interpret the *T*. *cruzi* cell cycle mRNA regulation, we compared the 305 transcripts with those regulated in the differentiation from **epimastigotes to metacyclic trypomastigotes,** published in a ribosome profiling study by our group [61]. In that study, we identified 1421 and 1323 genes upregulated at the level of mRNA abundance in epimastigotes and trypomastigotes respectively. We found that the 305-cell cycle regulated genes are 3.4-fold significantly enriched in the epimastigote stage, with a higher score in S-phase (Table 4). On the contrary, there is a significant absence of trypomastigote genes in the 305-gene set, as indicated by the 0.1- fold enrichment (P-value 3.4E-04) observed for S-phase genes. Since the epimastigote is a replicative developmental stage, while the trypomastigote is a non-replicative stage, the overrepresentation of epimastigote genes in the cell cycle regulated gene set is biologically consistent. In addition, when we looked at the 305 peaking genes in the ribosome footprint data of the same study, we also found a significant enrichment of epimastigotes actively translated mRNAs, which becomes particularly important at S phase (p-value 2.4 E-22).

**Table 4:** Analysis of cell cycle regulated genes during parasite development.

	Epimastigotes				Metacyclic Trypomastigotes			
	Up Transcriptome		Up Translatome		Up Transcriptome		Up Translatome	
Phase	O&(E)	FE (p-value)^a^	O&(E)	FE (p-value)^a^	O&(E)	FE (p-value)^a^	O&(E)	FE (p-value)^a^
G1	27 (11.0)	3.9 (2.0E-07)	27 (11.0)	5.5 (2.8E-10)	14 (10.2)	1.7 (7.4E-02)	7 (7.1)	1.0 (8.4E-01)
S	48 (15.0)	6.3 (2.8E-17)	48 (15.0)	8.6 (2.4E-22)	2 (14.4)	0.1 (3.4E-04)	4 (10.0)	0.4 (7.9E-02)
G2/M	28 (21.8)	1.6 (3.3E-02)	28 (21.8)	2.1 (1.3E-03)	19 (20.2)	1.0 (7.0E-01)	11 (14.2)	0.8 (7.7E-01)
All Reg	103 (46.1)	3.4 (1.4E-19)	103 (46.1)	4.6 (1.1E-17)	35 (48.9)	0.8 (2.7E-01)	22 (38.0)	0.6 (3.2E-02)

O&(E), observed and expected number of genes in each dataset; FE, Fold enrichment

^a^ Fold-enrichment and P-values obtained from Fisher contingency test.

### Analysis of compositional and structural features of the cell cycle regulated genes

**Fig 4:**
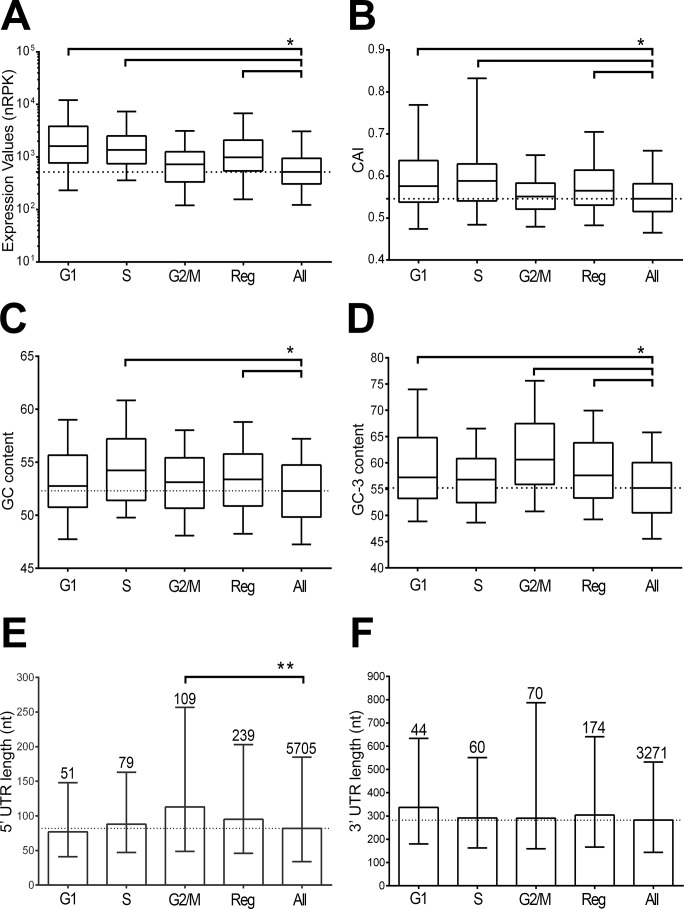
Analysis of structural characteristics of cell cycle regulated genes. Six parameters were calculated for five gene groups: phase specific cell-cycle genes at *G1 (70)*, *S (97)*, *G2/M (138)*, the 305 three phase combined gene dataset (*Reg*) and the total 7860 expressed genes (*All*). A. Gene expression levels of individual datasets. B. Codon adaptation index [54]. C, D. GC and GC3 content respectively. E, F. 5’ and 3’ UTR length respectively. The numbers above the bars indicate the number of UTRs that could be assigned to each group of genes. Box plots show the median and the 25–75 percentiles, whereas the whiskers represent the 5–95 percentiles. Bar graphs show the median and the interquartile range. Statistical significant differences were assessed by one-way ANOVA test and multiple comparisons were corrected by Bonferroni test (*, adjusted p-values lower than 0.01 by nonparametric Mann-Whitney test; **, exact p-value lower than 0.05).

In view of the potential importance of mRNA translational rates for cell cycle dependent transcripts that was underscored by the analysis of the ribosome footprint data [61], we decided to examine gene structural properties that are known to contribute to mRNA translation, including codon usage, GC content and UTR length. Overall, in exponentially growing epimastigotes cultures, cell cycle mRNAs are significantly more expressed than the average genome coding transcripts (Fig 4A). We firstly calculated their **codon usage**, which represent a well-established driver of translational rates [62]. As shown in Fig 4B, all the phase specific cell cycle regulated genes’ average CAIs are above the average CAI of the total expressed coding transcripts with more than 100 reads (denoted as “All”). When contrasting individual phases, it is evident that G2/M gene set has the lowest CAI, which resembles “All” genes. In addition, a group of S-phase and G1-phase genes have a well-adapted codon usage, which is showed by the extent of the upper whiskers of the box plot. Globally, the CAI correlates with the level of expression of the cell cycle genes. However, G1-phase genes show the highest median expression, whereas S-phase genes the highest CAI (Fig 4B).

In addition, it is proposed that the coordinated regulation of genes may in part rely on UTR length and base composition [63]; therefore, we investigated if the 305 peaking transcripts have any particular biases in these properties. In fact, the examination of the **G+C** and the GC3 content of the cell cycle regulated genes showed a statistically significant increase from the average G+C content of the mRNAs expressed in epimastigotes (Fig 4C). G+C deviation is reliant on the S genes trend, whereas GC3 deviation is mostly due to the trend of G2/M genes (Fig 4D).

Since there was not previous annotation of *T*. *cruzi* UTRs, we first determined the length of the UTRs of the sequenced mRNAs. For that purpose, reads containing the mini-exon sequence (ME) and poly-adenylated (PA) oligomers were mapped to the genome (S4 Fig). Only reads that mapped on inter-CDS regions with a mapping quality greater than 0 were retained. A total of 2251093 and 849487 reads were used for the 5’ UTR and 3’ UTR determinations respectively. Collapsing reads that corresponded to the same trans-splicing site rendered a total of 14649 5’ UTRs distributed among 6678 genes (an average of 2.2 UTRs per gene). We determined a total of 13362 3’ UTRs for 3718 genes (an average of 3.6 UTRs per gene). When comparing the length in base pairs for the obtained UTRs we observed a geometric mean of 80.8nt for 5’ UTRs, which is the exact length of the 5' UTR calculated previously in *T*. *cruzi* by Brandao *et al*. using 173 UTRs and different from the 35nt length proposed by Campos *et al*. [64, 65]. For the 3’ UTR, we found a mean length of 276.6nt, which is again closer to the 334nt reported by Brandao *et al*. than the 137nt median found by Campos *et al*. Then, we analyzed the length of the UTRs of the cell cycle regulated transcripts determined by our method (total of 5705 5’ UTRs and 3271 for 3’ UTR). We observed that cell cycle dependent genes have longer 5' UTR (median 95 vs 82nt) and 3' UTR (median 304 vs 282nt) relative to the average mRNAs coded in the genome (Fig 4E and 4F). These differences are statistically significant for the 5' UTRs of genes peaking at G2/M (113 vs 82nt).

Exceptionally among eukaryotes, the trypanosomatids organize and transcribe their genes in large **polycistronic** clusters [28]. Evidence obtained by analysis of the *T*. *brucei* cell cycle transcriptome [30] allowed Kelly *et al*. [66] to propose that the position of a gene in the polycistron contributes to its transcript abundance throughout the cell cycle. In order to assess if that is also the case for the *T*. *cruzi* cell cycle genes, we determined the position of the genes in their cognate polycistrons. We observed that cell cycle genes tend to be further away from the TSS in comparison to the average genes (63 vs 57 kb, p-value <0.06). Specifically, G1, S and G2/M distances to the TSS are 64, 60 and 66 kb respectively. Thus, the longest distance is seen for G2/M genes, a finding that goes in agreement with *T*. *brucei*'s [66]. In fact, when we performed the analysis done by Kelly *et al*. [66] using the *T*. *cruzi* information instead, we found a similar proportionality between cell cycle gene expression and the location of the genes in the polycistron, where mRNA abundance increases with the distance to the polycistron start for G2/M, and decreases for S-phase genes (S3 Fig).

### Analysis of potential cell cycle regulatory RNA motifs in cell cycle regulated genes

**Fig 5:**
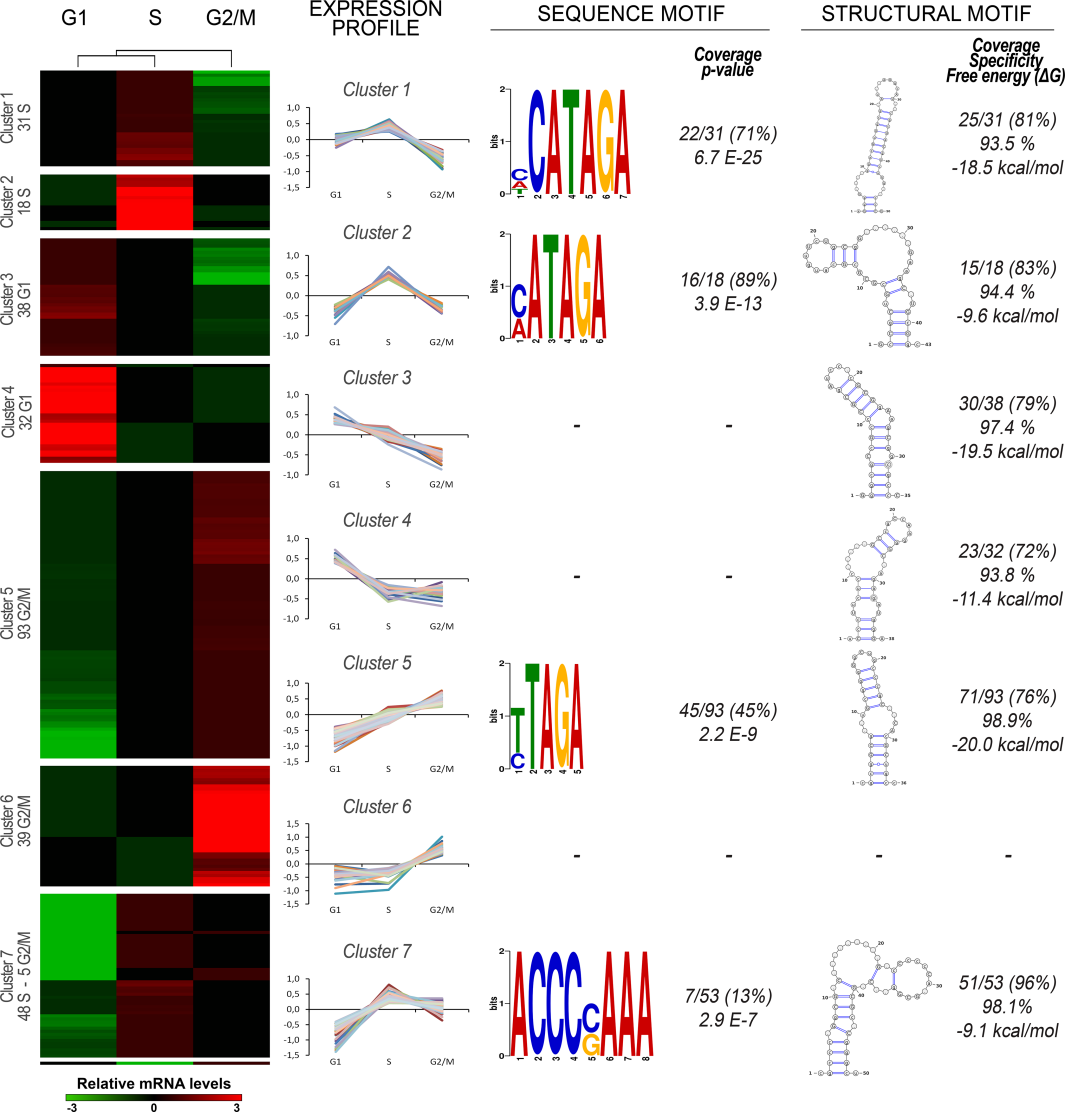
Prediction of RNA motifs in cell cycle co-regulated gene clusters. Left panel: Heatmap representation of the expression of 305-cell cycle genes. The gene expression values (log_2_ normalized read counts) were subjected to a clustering analysis in the GENE-E software (Broad Institute). Both columns (samples) and rows (genes) were clustered by Pearson correlation. Right panel: the gene expression profile in the three sequenced cell cycle phases was plotted for each cluster. Consensus sequence motifs found exclusively at the 3’ UTRs are presented right to each cluster, and the coverage and the p-value obtained from the DREME search is indicated. Consensus structural motifs found with CMfinder are presented on the right most column, and their coverage, specificity and free energy are indicated. The structural motifs were all found at the 5 ‘UTR except for the motif found in cluster 7. None of the motifs found in cluster 6 reach the thresholds. The best motif for each cluster is depicted.

Due to the absence of transcriptional control in the trypanosomatids, the regulation of mRNA processing, decay and translatability is thought to have a relevant role in gene expression regulation. To investigate the presence of sequence elements in the cell cycle genes we searched for overrepresented sequences in the mRNAs' UTRs, since they are known to contain the majority of the RNA motifs affecting the post-transcriptional regulation of the genes. In order to identify groups of co-regulated transcripts with near identical expression patterns along the three cell cycle phases sequenced here, a non-parametric clustering of the 305 cell cycle regulated genes was carried out, which allowed the definition of 7 clusters (heatmap on Fig 5). We found six phase specific clusters (two per phase) and one that combines genes of S and G2/M. One gene (TcCLB.511805.20, Trichohyalin) remained ungrouped in this analysis, being the only gene strongly down-regulated in S phase compared to G1 and G2/M.

We then used the DREME tool (from MEME suite) to search for enriched motifs within each cluster. We did not find any enriched RNA sequence at the 5' UTR of the seven gene clusters. However, we found at least two overrepresented RNA sequences in the 3' UTRs. The top-ranking motif is the seven-nucleotide sequence c/a/tCAUAGA, which is significantly overrepresented in the S-phase cluster 1 (p-value 6.7E-25), being borne by 22 of the 31 genes (Fig 5). In addition, a c/aAUAGA motif, whose sequence is contained in the previous one, is also significantly enriched in the S-phase cluster 2 (16 out of 18 genes). Of note, a four nucleotide RNA element, UAGA, also contained in the previous motifs, is overrepresented in G2/M-phase cluster 5; however, the significance of the enrichment and the proportion of genes with the motif are lower than those described for the S-phase clusters. These three motifs are variants of the octamer consensus sequence called the “cycling control sequence” element (CS), present in several cell cycle dependent mRNAs, which was originally described in *Crithidia fasciculata* [67] and later found also in *Leishmania major* [68] and *T*. *cruzi* orthologous of *T*. *brucei* cell cycle regulated genes [30]. In *T*. *cruzi*, this motif is located almost exclusively at the 3' UTR of the 305 cell-cycle regulated transcripts, where it is found at an average distance of 148 bp from the end of the translated region. Fourteen percent of the genes (5/36) have more than one motif at their 3’UTR, with one gene bearing 3 motifs (TcCLB.509151.130, a dUTP diphosphatase). In addition, we found 378 genes with CS-elements at their 3’UTR of the total 7860 expressed genes. Interestingly, the 305-cell cycle regulated genes are 5-fold enriched in the former group (56/378 genes, fisher test, p-value 6.4 E-19). Finally, an eight-nucleotide sequence ACCCc/gAAA shows a smaller but significant enrichment in cluster 7, being borne by only 7 genes (13). For the *T*. *cruzi* orthologues of the already described CS-element containing genes (as TOP2, KAP3, DHFR-TS, RPA1), we looked at the presence of this sequence in the experimentally defined UTRs (S5 Fig). Contrary to what is described in *C*. *fasiculata* and *L*. *major*, the 5’ UTRs of DHFR-TS and KAP3 do not bear the CS-element. Indeed, KAP3 does not present a CS-element within its 3’ UTR either. But the remaining UTRs present at least one CS-element, and TOP2 presents 4 elements on its long 5’-UTR, as has been described in the other species.

Since RNA transcript regulatory elements can rely on a structure instead of an RNA sequence, we also searched for structural motifs in the UTRs of the co-regulated groups of genes. We obtained the top 3 conformational motifs for each co-regulated cluster using *CMfinder*. Then, we further selected them based on a threshold of specificity greater than 90%, ΔG lower than -8.0 kcal/mol and coverage greater than 50%. The top ranked motif for each cluster is presented on the right most part of Fig 5 (see S6 Table for the complete list of motifs). Interestingly, all the motifs, except for cluster 7’s motif, are located at the 5’ UTRs. These structural signals are stem-loops interrupted by one to two bulges of various lengths.

## Discussion

In the present study, we analyzed the changes in mRNA abundance throughout three stages of the cell cycle of *T*. *cruzi* epimastigotes, seeking to identify gene regulatory patterns and specific genes involved in parasite proliferation. In order to obtain cultures enriched in different cell cycle phases, we used a **hydroxyurea synchronization** protocol previously developed for this parasite, obtaining a 70% enrichment in synchronic G1, S and G2/M cell cultures. Although this method has been extensively used in cell cycle studies of many organisms, it has the disadvantage of producing chemically perturbed cell cycle phases; therefore, it can be expected that some of the findings may not reflect the physiological cell cycle [69]. Unfortunately, since synchronic *T*. *cruzi* cultures lose synchrony after one division, the second cell cycle after HU release is not suitable for cell cycle studies. Nevertheless, a vast amount of literature indicates that the cycle after hydroxyurea arrest is comparable to the natural cycle [70]. Specifically, the study of the cell cycle transcriptome of *T*. *brucei*, obtained with a protocol of serum starvation (perturbed cycle) or an elutriation method (unperturbed cycle), demonstrated a vast similarity in transcript regulation amplitude (80% match) and expression chronology (72% match) between the two methods [30]. In addition, alternative protocols like elutriation or cell sorting, which are presumed to produce “unperturbed” cell cycle phases, are known to introduce alterations in gene expression [71, 72]. Finally, cell sorting of *T*. *cruzi* using permeant dyes is rarely reported (and for limited application only [73, 74]) in trypanosomatids literature, and would not supply sorted cell numbers adequate for RNA collection for deep sequencing. Therefore, HU synchronization is currently a method of choice for transcriptomic studies of the cell cycle in trypanosomatids.

The high reproducibility of the synchronization protocol used in our study, confirmed by RT-qPCR of individual replicates, allowed us to **sequence** pooled equimolar triplicates of each cell cycle stage. The global comparison of the three phase specific datasets indicates that the transcriptome is only slightly modified along the cell cycle. Indeed, in contrast to the >0.9 correlation coefficients among the three cell cycle transcriptomes, developmental changes throughout the *T*. *cruzi* life cycle have shown a massive transcriptome remodeling, accounting for Pearson correlations of 0.5–0.6 for the epimastigote-trypomastigote transition [55, 61]. Thus, our finding supports the hypothesis of small intra-stage transcriptomic changes that was recently proposed by Li *et al*. [55]. Yet, we identified a list of **305 coding gene transcripts** that significantly change their abundance during the three cell cycle transitions analyzed (1.5-fold, p-value<0.01). They are enriched in **GO terms** that correctly delineate the central processes of the corresponding cell cycle stage, which indicates that our approach allowed for a reliable identification of periodically regulated mRNAs. In addition, the identification of several transcripts for **proteins** that were already demonstrated to be periodically expressed in trypanosomatids, provides an independent validation of our study. These proteins include histones [17], cyclins [19], proliferative cell nuclear antigen [75], kinetoplast proteins DHFR, TOP2 [67] and KAP3 [76], Puf9 [77], polo kinase TbPLK orthologue TcCLB.506513.160 [78], and kinetochore associated protein 1 KKT1 orthologue, TcCLB.507641.190 [79]. Other proteins, like TcHMGB [80] and putative chromosomal passenger protein TcCPC2, Tc00.1047053506221.110 [81], are known to participate in the cell cycle but their periodical expression has not been demonstrated yet. It is worth mentioning that we do not find basal body and flagellar proteins regulated in the 305 periodical genes, in contrast to the cases of *T*. *brucei* and *Leishmania* [30]. That might indicate divergent levels of gene expression regulation of cell cycle genes with this ontology or divergent cellular mechanism for organelle segregation, as has been proposed before [82]. Additionally, the identification of several kinetoplast specific periodically expressed genes supports the coordination between the nuclear and kinetoplast replication in *T*. *cruzi* that has been previously suggested [83].

When we compared the periodically expressed proteins of the two human **parasitic trypanosomatids,** we found a relatively low coincidence between them (30% of transcript identity and only 39% of synchronic expression). Although differences in the methods of parasite isolation, as well as differences in data processing, might account in part for the observed dissimilarities, the biology of the two parasites may also contribute to them. In fact, the two organisms differ in relevant features, such as chronology and structural organization of events along the cell cycle [5, 82]. Importantly, *T*. *cruzi* replicates intracellularly (vertebrate cells) and extracellularly (insect), whereas *T*. *brucei* only proliferates in the latter way. Unlike what has been described for *T*. *brucei*, the replication of the kinetoplast in *T*. *cruzi* epimastigotes starts later than the replication of the nucleus, and the kinetoplast divides at late nuclear G2. Likewise, the elongation of the daughter flagellum and the division of the flagellar pocket occur later in *T*. *cruzi* and the new flagellum does not grow attached to the old one or to the cell body [5]. Additional diversity between the parasites resides in the nuclear architecture of the DNA synthesis during S phase [84]. Striking differences between the two species in the structure of the sub-telomeric regions are also expected to contribute to the divergence in nuclear division [discussed in 83]. Finally, the distinct response of the two species to the cell cycle arrest induced by hydroxyurea suggests regulatory differences between the parasites [85].

Given the therapeutic importance of the identification of proliferative differences between the parasite and the **host**, we contrasted the periodically expressed genes of both species. We showed that most of the *T*. *cruzi* cell cycle transcript (62%) do not have homologues in man. In addition, the low proportion of orthologues that are commonly regulated in both species (24%) points to the divergence of the molecular mechanism driving cell cycle or the divergence in the level of cell cycle transcripts regulation used in each organism.

The focus of the present study was not only to identify genes periodically expressed in epimastigotes of *T*. *cruzi*, but also to identify putative global gene expression regulatory patterns that operate on these transcripts. Seeking for an insight on further transcript regulatory steps, we looked at the distribution of our gene set in the **nuclear-cytoplasmic** RNA-seq and ribosome profiling data on *T*. *cruzi* exponentially grown epimastigotes [60, 61]. The finding of an overrepresentation of cell cycle transcripts at the cytosol indicates that periodically expressed transcripts are probably actively maintained in the cytosol in replicating epimastigotes. We also found that cell cycle transcripts are significantly overrepresented in the actively translated mRNA fraction, with emphasis at S phase and decrease at G2/M. Therefore, periodically expressed mRNAs may be also controlled at the level of translatability. An assessment of the translational control of the cell cycle phases has been recently done by Stump *et al*. [86], who discovered a widespread translational regulation of molecular complexes involved in cell cycle progression in human cells. More recently, the reduction of mRNA translatability of a set of 200 gene transcripts during the G2/M transition of mammalian cell cycle has been demonstrated by ribosome footprint, but most of them do not change their total mRNA abundance [87]. The authors suggest that gene specific mRNA translatability complements other post-translational mechanisms to establish the mitotic proteome.

We then investigated if the codon composition of the cell cycle genes could be contributing to their **translatability** as inferred from the ribosome footprint data. We found that periodically expressed genes have CAIs higher than the average for the genome, although their median CAI are barely above 0.5. This suggests that codon adaptation might be globally contributing to the higher translational efficiency of periodically expressed genes relative to that of the average genome. However, the CAIs of the 305 genes are still low, which indicates their preference for non-optimal codons. There is only one study of codon usage along the cell cycle, but it investigates codon preferences in terms of codon-anticodon binding affinities [88]. It found that periodically expressed genes have specific codon preferences for non-optimal codons in four distant species. In particular, G1 genes are shown to be biased to optimal codons. In addition, they demonstrate that tRNA concentration inversely follows the change in codon preferences in the different phases. Although their results are not directly comparable to ours, we show that a group of S and G1 peaking genes have high CAIs. Nevertheless, the relative higher expression of G1 genes in the absence of a relatively higher CAI average indicates that additional forces, like tRNA concentration, ATP availability, structural and sequence motifs at the UTRs, might be acting on their expression.

In light of previous awareness about the association between gene expression and structural gene features, we investigated several structural properties of the cell cycle genes. The **G+C** content of the genes has been linked with the level of expression in some reports, however the relevance of these findings is controversial [89]. The fact that *T*. *cruzi* 305 periodically expressed genes are slighter richer in G+C and GC3 and show higher CAIs and transcript expression level than randomly selected genes, supports that base composition might be contributing to gene expression in this case. There is also mounting evidence showing that the length of the mRNA, in particular the UTRs, affects gene expression [90–92]. Our data points out the presence of significant longer UTRs in the G2/M genes. Interestingly, G2/M genes have been shown to be translationally silenced [87] and the long 5' UTR has been related to translational inhibition [92]. Many growth-related mRNAs have unusual 5′ UTRs, which are both long and GC-rich, conditions that promote the formation of stable secondary structures or increase the chances to harbor upstream open-reading frames (uORFs) or internal ribosome entry sites (IRESs). This aspect might be an interesting subject of further investigation.

In addition, our study of the dependence of cell cycle gene expression on the **position of the genes along it cognate polycistron** supports previous findings in *T*. *brucei* [66], thus providing further empirical evidence for the importance of the position of the transcript inside the polycistronic unit in trypanosomatids. It reinforces the concept that gene position is actively favoring the maintenance of syntheny in these organisms, despite their long evolutionary distance.

The coordinated regulation of groups of transcripts can be achieved by the use of common **nucleotide motifs**, which can be recognized by RNA binding proteins (RBPs), which in turn modulate the fate of the RNA in terms of processing, stability and sub-cellular location [93]. When we search for sequence signals at the UTRs, we only found enriched RNA sequence motifs at the 3' UTRs, the region which is thought to hold the majority of the cis-elements that modulate mRNA abundance [94, 95]. The most enriched motif found here is a variant of the CS-element, previously characterized in cell cycle genes. The high coverage of the motif in the 3' UTR of the 305 genes indicates that *T*. *cruzi* might strongly rely on this sequence for periodically gene expression regulation. Several CS-element binding proteins (CSBPs) have been identified and characterized as cell cycle regulators in *Crithidia fasciculata* [96], however the molecular mechanism of their action is not completely understood.

Finally, several putative stem-loops at the UTRs of the clusters of co-regulated genes were identified. Their high specificity together with their high coverage suggests their potential use in gene regulation as RNA **structural motifs.** Moreover, their preference for the 5' UTR indicates a role related to translation which remains to be investigated.

## Conclusions

We have identified 305 cell cycle regulated mRNAs in proliferative epimastigotes of *T*. *cruzi* using the most sensitive experimental approach currently available. The enriched GO terms of the cell cycle differentially expressed genes strongly supports their cell cycle-specific functions. In addition, our study assigns a cell cycle regulatory role for several hypothetical and previously unstudied proteins. The identification of the molecular networks driving parasite specific proliferation is an important step towards the design of improved chemotherapies to treat Chagas disease. It might also facilitate the characterization of parasite specific individual proteins.

In the absence of transcriptional control of gene expression, trypanosomatids constitute an interesting model to discover new patterns of post-transcriptional regulation of gene expression. In this scenario, our results strongly indicate that the periodically expressed mRNA transcripts are actively subject to control at least at three levels: nuclear-cytosolic distribution, RNA abundance and translatability. They also indicate that genome positioning, nucleotide composition, mRNA length, as well as specific sequence and conformational elements at the UTRs, contribute to the periodical gene expression in *T*. *cruzi*. The multi-step control of gene expression of proteins involved in a specific biological process has been scarcely analyzed in the literature, thus our study shows how functional genomics can be used to uncover integrated RNA transcript control mechanisms.

## Supporting information

S1 Fig Assessment of sequence depth threshold for reliable gene count. (DOCX)

S2 Fig RT-qPCR validation of cell cycle regulated genes. (DOCX)

S3 Fig Influence of the gene localization in the polycistronic unit on cell-cycle expression. (DOCX)

S4 Fig Determination and length distribution of the UTRs. (DOCX)

S5 Fig Localization of the CS-element in *T*. *cruzi* in comparison to previously studied trypanosomatids. (DOCX)

S1 Table Sequencing and Mapping data of *T*. *cruzi* epimastigote cell cycle phases. (XLSX)

S2 Table Primer sequence and PCA input data (normalized ΔΔCt and read counts). (XLSX)

S3 Table Pearson correlation of individual replicates and pooled RNA-Seq samples of *T*. *cruzi* epimastigote cell cycle phases. (XLSX)

S4 Table Read ncounts of G1, S and G2/M *T*. *cruzi* cell cycle phases and list of DEGs. (XLSX)

S5 Table GO enrichment analysis for the cell cycle peaking genes. (XLSX)

S6 Table RNA structural motifs on clusters of co-regulated cell cycle genes. (XLSX)
