# Supplementary material for: Transcriptome-wide analysis of the Trypanosoma cruzi proliferative cycle identifies the periodically expressed mRNAs and their multiple levels of control
Source: PLoS One. 2017 Nov 28;12(11):e0188441. doi: 10.1371/journal.pone.0188441 (PMC5705152; doi:10.1371/journal.pone.0188441)
Supplement: S1 Fig — (DOCX) [file pone.0188441.s001.docx]

# Supplementary Figure 1


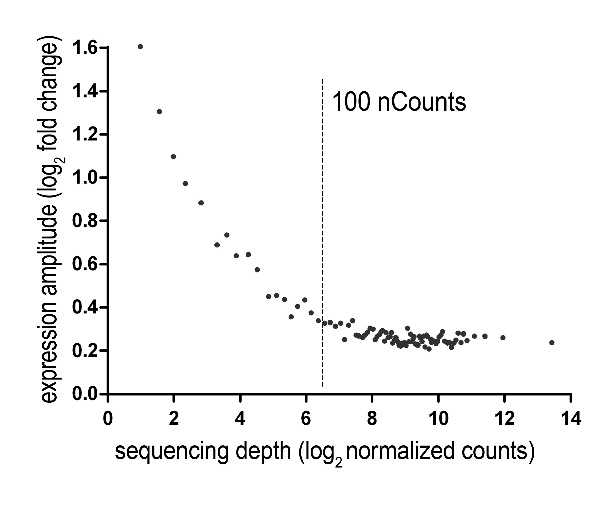


**Assessment of sequence depth threshold for reliable gene count.**

Gene expression amplitude establishment as a function of sequencing depth. Gene expression amplitude of individual genes was calculated as the fold change between the minimum and maximum expression value observed, combining the data of the three cell cycle stages. After sorting the genes by their minimum read count, the median of the amplitude was calculated for a moving window of 50 genes, and was then plotted against the median of the minimum read count registered in the same window (sequencing depth), performed previously performed for the *T. brucei* cell cycle transcriptome (Archer SK et al., 2011). The vertical dashed line indicates the location of the 100nCounts threshold in the plot.
