# Supplementary material for: Transcriptome-wide analysis of the Trypanosoma cruzi proliferative cycle identifies the periodically expressed mRNAs and their multiple levels of control
Source: PLoS One. 2017 Nov 28;12(11):e0188441. doi: 10.1371/journal.pone.0188441 (PMC5705152; doi:10.1371/journal.pone.0188441)
Supplement: S2 Fig — (DOCX) [file pone.0188441.s002.docx]

# Supplementary Figure 2


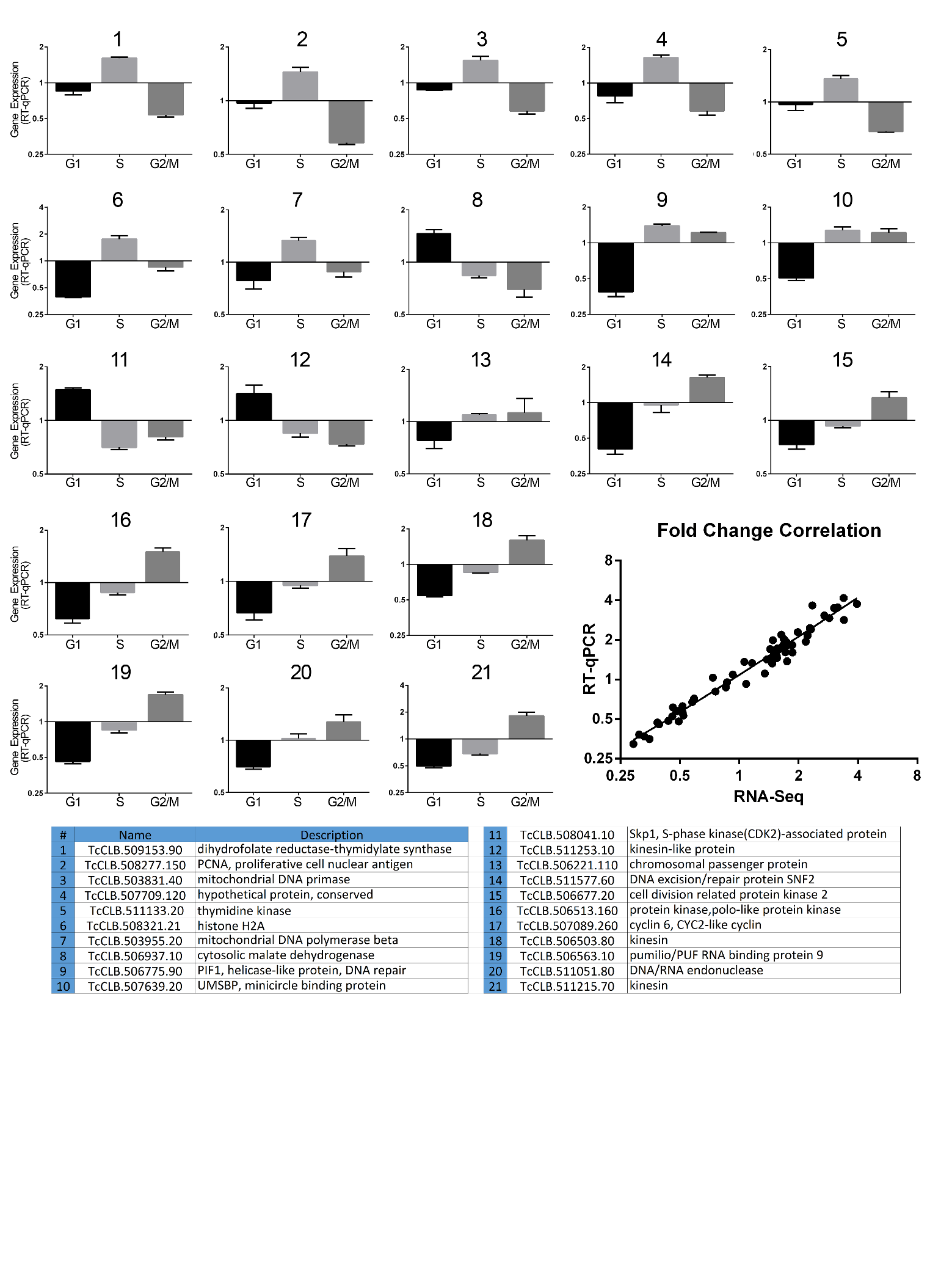


**RT-qPCR validation of cell cycle regulated genes.**

The three independent synchronization experiments used to prepare the pooled samples sequenced were individually analyzed by RT-qPCR to validate 21 differentially expressed genes. The mRNA levels were normalized to the Tubulin housekeeping gene and are presented in log2 scale. Bar charts represent mean changes (± SEM) in gene expression relative to the average of expression of each gene in the three cell-cycle stages determined by the ^–ΔΔCt^ method. The box plot shows the fold change in expression for the three cell cycle transitions (G1/S, S/G2 and G2/G1) assessed by RT-qPCR (average ΔΔCt of three independent replicates of each phase) and RNA-Seq using DESeq (normalized reads of pooled triplicates). The inset table presents the Tritryp gene IDs and the gene description.
