# Supplementary material for: Transcriptome-wide analysis of the Trypanosoma cruzi proliferative cycle identifies the periodically expressed mRNAs and their multiple levels of control
Source: PLoS One. 2017 Nov 28;12(11):e0188441. doi: 10.1371/journal.pone.0188441 (PMC5705152; doi:10.1371/journal.pone.0188441)
Supplement: S3 Fig — (DOCX) [file pone.0188441.s003.docx]

# Supplementary Figure 3

**Influence of the gene localization in the polycistronic unit on cell-cycle expression**. In this analysis, a sliding window of 40 kbp was moved in 1 kbp steps in the direction of transcription, away from the determined transcription initiation sites. In each step the mean mRNA abundance of all genes occurring within the 40 kb window (across all transcription units) was calculated for each cell cycle stage. The fold change for each cell cycle phase versus the mean of all three phases was calculated and plotted versus the distance from transcription initiation site. Windows containing fewer than 200 genes were not considered for this study.
