# Supplementary material for: Transcriptome-wide analysis of the Trypanosoma cruzi proliferative cycle identifies the periodically expressed mRNAs and their multiple levels of control
Source: PLoS One. 2017 Nov 28;12(11):e0188441. doi: 10.1371/journal.pone.0188441 (PMC5705152; doi:10.1371/journal.pone.0188441)
Supplement: S4 Fig — (DOCX) [file pone.0188441.s004.docx]

# Supplementary Figure 4


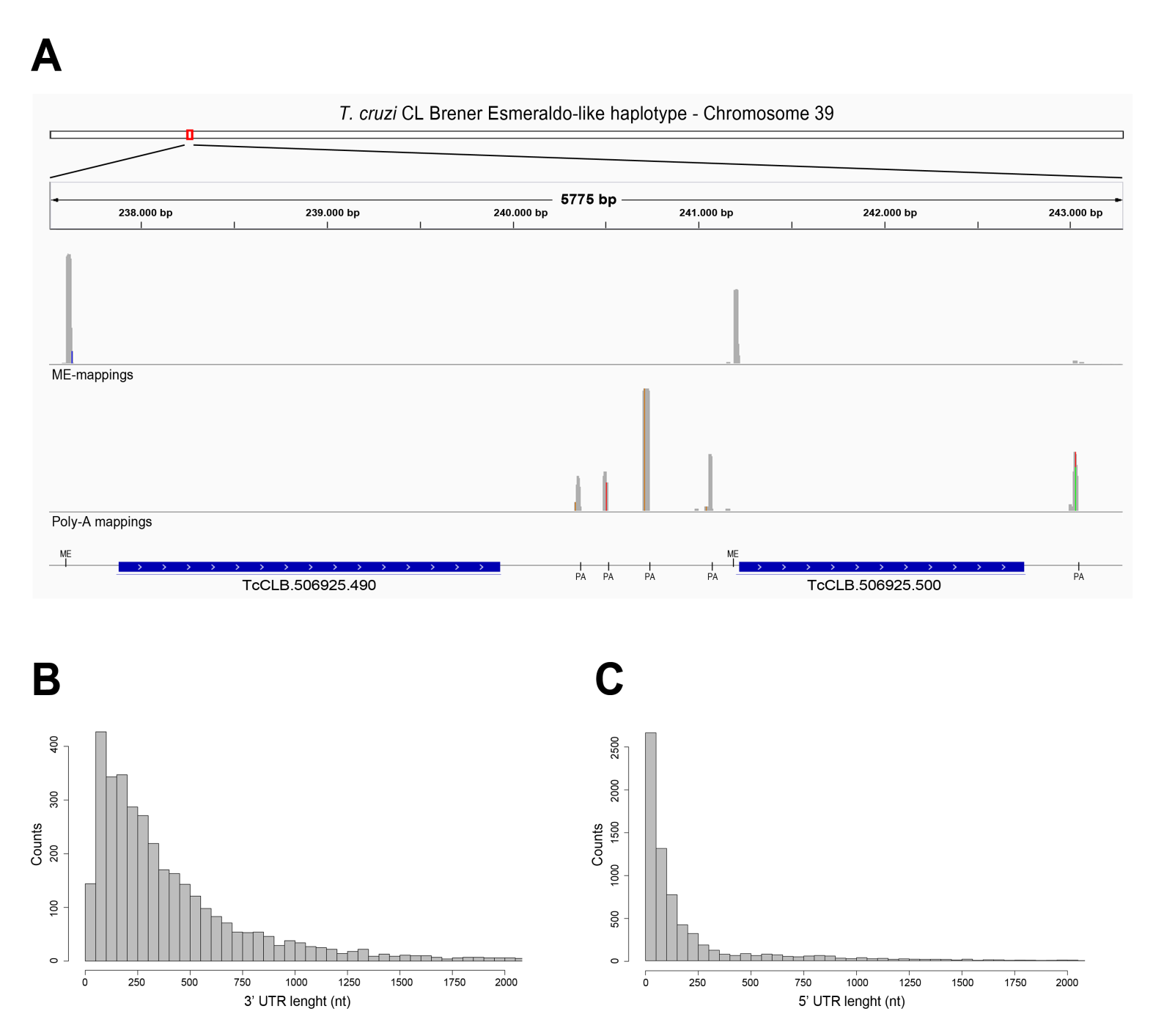


**Determination and length distribution of the UTRs.** The UTRs of the transcripts sequenced in this study were annotated by mapping the mini-exon (ME) and poly-A (PA) sites using an in-house method. A. Schematic representation of the mapping results for an example region as depicted in the IGV genome browser. B and C. Histogram representation of the 5´ and 3´UTR size distribution respectively for all UTRs annotated.
