# Supplementary material for: Transcriptome-wide analysis of the Trypanosoma cruzi proliferative cycle identifies the periodically expressed mRNAs and their multiple levels of control
Source: PLoS One. 2017 Nov 28;12(11):e0188441. doi: 10.1371/journal.pone.0188441 (PMC5705152; doi:10.1371/journal.pone.0188441)
Supplement: S5 Fig — (DOCX) [file pone.0188441.s005.docx]

# Supplementary Figure 5

#
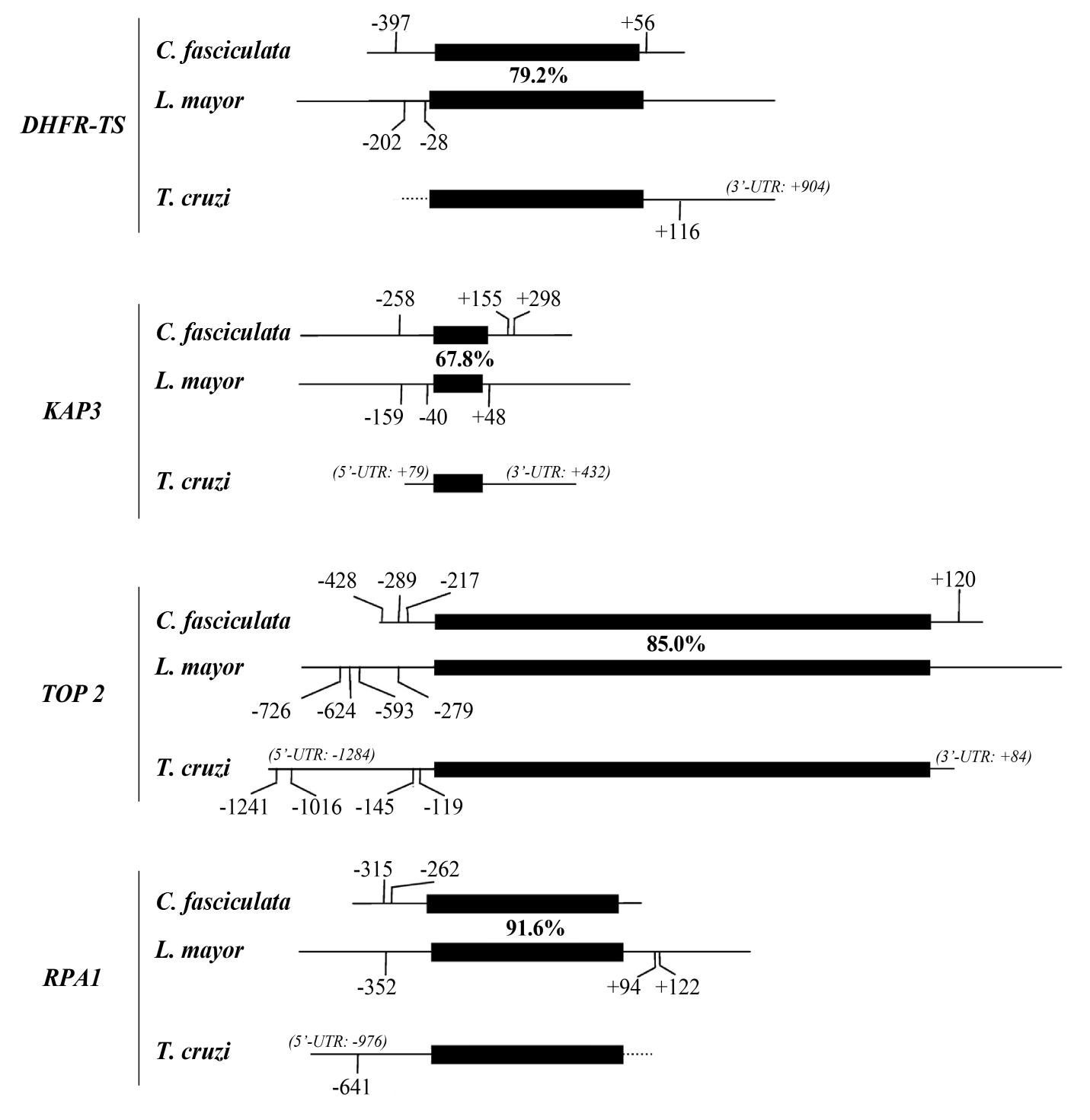


**Localization of the CS-element in *T. cruzi* and comparison to previously studied trypanosomatids.** Figure 1, from Zick *et al*, 2005 was modified to include the description of the CS-element on the *T. cruzi* orthologues genes. The motif was searched on the experimentally determined UTRs whenever possible (the UTR size is presented in brackets). For DHFR-TS and RPA1, we were not able to annotate the 5’-UTR and 3’-UTR respectively, and the motif search was made on an arbitrary UTR sequence consisting on 100 bp upstream of the AUG codon for DHFR-TS and 100 bp downstream of the STOP codon for RPA1 (in both cases represented as a dotted line). We did not extend those two UTRs because the next gene was located closer than 300 bp in both cases.
